# Incidence and risk factors associated with preoperative deep venous thrombosis in the young and middle-aged patients after hip fracture

**DOI:** 10.1186/s13018-021-02902-8

**Published:** 2022-01-11

**Authors:** Kai Ding, Haicheng Wang, Yuxuan Jia, Yan Zhao, Weijie Yang, Wei Chen, Yanbin Zhu

**Affiliations:** 1 Department of Orthopaedic Surgery, The 3rd Hospital of Hebei Medical University, No. 139 Ziqiang Road, Shijiazhuang, 050051 Hebei People’s Republic of China; 2 Key Laboratory of Biomechanics of Hebei Province, Shijiazhuang, 050051 Hebei People’s Republic of China; 3 Orthopaedic Institution of Hebei Province, Shijiazhuang, 050051 Hebei People’s Republic of China; 4 NHC Key Laboratory of Intelligent Orthopeadic Equipment (The Third Hospital of Hebei Medical University), Shijiazhuang, People’s Republic of China; 5 School of Nursing, Hebei Medical University, Shijiazhuang, 050000 Hebei People’s Republic of China; 6 Department of 2017 Clinical Medicine, School of Class 4, Basic Medicine, Hebei Medical University, Shijiazhuang, Hebei Province People’s Republic of China

**Keywords:** Deep vein thrombosis, Young and mid-aged, Hip fracture, Epidemiology, Perioperative management

## Abstract

**Objective:**

This study aims to investigate the incidence, occurrence timing and locations of preoperative DVT and identify the associated factors in this group.

**Methods:**

A retrospective analysis of collected data in young and middle-aged (18–59 years) patients who presented with hip fracture between October 2015 and December 2018 was conducted. Before operation, patients were routinely examined for DVT by Duplex ultrasonography (DUS). Electronic medical records were retrieved to collect the data, involving demographics, comorbidities, injury and laboratory biomarkers after admission. Multivariate logistic regression analysis was performed to identify factors that were independently associated with DVT.

**Results:**

Eight hundred and fifty-seven patients were included, and 51 (6.0%) were diagnosed with preoperative DVT, with 2.5% for proximal DVT. The average age of patients with DVT is 48.7 ± 9.4 year, while that of patients without DVT is 45.0 ± 10.9 year. The mean time from injury to diagnosis of DVT was 6.8 ± 5.5 days, 43.1% cases occurring at day 2–4 after injury. Among 51 patients with DVT, 97 thrombi were found. Most patients had thrombi at injured extremity (72.5%), 19.6% at uninjured and 7.8% at bilateral extremities. There are significantly difference between patients with DVT and patients without DVT in term of prevalence of total protein (41.2% vs 24.4%, *P* = 0.008), albumin (54.9% vs 25.6%, *P* = 0.001), low lactate dehydrogenase (51.0% vs 30.3%, *P* = 0.002), lower serum sodium concentration (60.8% vs 29.9%, *P* = 0.001), lower RBC count (68.6% vs 37.0%, *P* = 0.001), lower HGB (51.0% vs 35.1%, *P* = 0.022), higher HCT (86.3% vs 35.1%, *P* = 0.022) and higher platelet count (37.3% vs 11.3%, *P* = 0.001). The multivariate analyses showed increasing age in year (OR 1.04, 95% CI; *P* = 0.020), delay to DUS (OR, 1.26; *P* = 0.001), abnormal LDH (OR, 1.45; *P* = 0.026), lower serum sodium concentration (OR, 2.56; *P* = 0.007), and higher HCT level (OR, 4.11; *P* = 0.003) were independently associated with DVT.

**Conclusion:**

These findings could be beneficial in informed preventive of DVT and optimized management of hip fracture in specific group of young and mid-aged patients.

## Introduction

Among the elderly population, hip fracture is the most common and severe injury and also often the most extensive and in-depth research topic in the clinical studies. On one hand, the prevalent osteoporosis in such a population makes this injury a public concern the worldwide, relating to the current and approaching substantial health expenditure by the next several decades [1, 2]. On the other hand, the pre-existing comorbidities and the fragility in the elderly would compromise the treatment outcomes, and potentially cause 10–30% 1-year mortality after operation and approximately 33% partial or complete loss of independence [3–5]. With contrast, hip fractures in young and middle-aged population is not extensively and deeply studied, although they represent approximately one third of the over hip fractures in the adults [6].

Differing elderly patients, young and middle-aged patients had hip fractures caused by higher-impact mechanism, with higher bone fracture severity and soft tissue damage, which probably introduces a stronger immune response or inflammatory reaction post-trauma [7]. Therefore, we infer perioperative undesirable events relevant to hip fracture itself or secondary to systematic immune/inflammatory response in the young and middle-aged population might be different from in elderly patients. Deep venous thrombosis (DVT), an either thromboembolic event, can progressed proximally into pulmonary embolism (PE) and is related to mortality [8]. However, in most trauma subspecialties, the incidence of DVT was highly variable, and specific in hip fracture it was reported to be 10–65%, mainly depending on the anti-thromboembolic agent use, characteristics of included patients, extensive definitions of DVT or the study design [9, 10]. Furthermore, regarding the perioperative factors that may predict the incident DVTs, the literature displayed the inconsistent or even contradictory results [9–12]. It is well-established that, abundant knowledge of epidemiologic characteristics on DVTs, such as their incidence, timing, locations and the predictors, are of vital importance in risk assessment and stratification, facilitating targeted perioperative management of hip fracture. However, by far as we know, the attention on DVT after hip fracture specific in the young and middle-aged patients is not adequately focused, and the non-specific findings available in literature might not be applicable.

In this study, we focused on a subgroup of young and middle-aged patients presenting with hip fractures, with aims, (1) to examine the incidence rate of preoperative DVT; (2) to investigate the incidences over time and detailed locations of the DVTs, and (3) to identify some factors that could predict the incident DVT.

## Methods

We conducted this retrospective study in accordance with guideline of Strengthening the Reporting of Cohort Studies in Surgery (STROCSS). All data were extracted from database of Surgical Site Infection in Orthopaedic Surgery (SSIOS), where prospective method was used to collect data with presupposed aim to investigate surgical incision infection after orthopaedics surgeries for any bone-related diseases (trauma, degenerative diseases or bone tumor). Informed consent was signed by all the participants. We included a total of 857 young and middle-aged patients of hip fracture admitted from October 2015 and December 2018.

### Inclusion and exclusion criteria

The inclusion criteria were: (1) young and middle-aged (18–59 years) patients; (2) hip fractures (femoral neck or intertrochanteric fracture); (3) and patients with complete data. The exclusion criteria were: (1) age outside the predefined range; (2) open fractures or old fractures (> 14 daays from injury); (3) pathological or metastatic fractures; (4) multiple trauma or concurrent fractures; (5) active cancer; (6) history of DVT or pulmonary embolism; (7) recent thromboembolism therapy (such as aspirin, warfarin, heparin, low molecular weight heparin or others) within 3 months.

### Diagnosis and classification of DVT

DVT was diagnosed in accordance with the Guidelines for the Diagnosis and Treatment of Deep Vein Thrombosis proposed by Chinese Medical Association [13]. According to institutional policy, hip fracture patients are asked to receive duplex ultrasonography (DUS) examination for potential DVT of the bilateral extremities at admission, subsequently every 3–7 days and when any symptoms suggestive of DVT presented, and the veins involved may be any one or any combined (common femoral vein, superficial femoral vein, deep femoral vein, popliteal vein, anterior tibial vein, posterior tibial vein or peroneal vein). The criteria for DUS diagnosis of DVT are: loss of or non-compressibility, lumen obstruction or filling defect, lack of respiratory variation in above-knee vein segments and inadequate flow augmentation to veins of calf and foot with compression maneuvers. Based on DUS results, the patient would be given therapeutic or prophylactic thromboembolic agents, thereafter, second or further DUS scans are conducted until the operative procedure.

Depending on the location of the thrombus, we classified patients into two groups: distal DVT group where patients had any thrombus at anterior tibial vein, posterior tibial vein, peroneal vein solely or combined, and proximal DVT group where patients had any thrombus at popliteal or femoral vein, regardless of having co-existing distal DVTs. It is of note, isolated thrombosis solely located in the intramuscular veins (e.g. soleal or gastrocnemius vein) is excluded from this study, due to their less clinical significance [14].

### Data collection and definitions of specific parameters

All the data were extracted from the patients' hospitalization medical records, related to demographics (age and gender), body mass index (BMI: normal ≤ 23.9; overweight 24.0–27.9; obesity ≥ 28), smoking, alcohol drinking status, comorbidities (hypertension, diabetes, heart disease, cerebrovascular disease, pulmonary disease, liver disease, renal insufficiency), injury-related data (fracture type, time from injury to DUS scan) and the laboratory results (platelets, fasting blood glucose (FBG), total protein, albumin, lactic dehydrogenase (LDH) level, hypersensitive C-reactive protein (HCRP), red blood cell (RBC), hemoglobin, white blood cells (WBC), neutrophils. lymphocytes, sodium concentration, alanine transaminase (ALT), aspartate aminotransferase (AST), uric acid (UA), red cell distribution width (RDW), platelet distribution width (PDW), plasma D-dimer).

Smoking and alcohol drinking status were determined based on their current status documented in patients' medical records, which were reported by the patients, guardians or relatives. Presence or not of comorbidities was determined according to patient s' self-reported history (e.g. cerebrovascular accident, myocardial infarction) or chronic persistent state for a certain condition (e.g. hypertension, diabetes, liver disease, renal disease, et al.). Fracture type was classified based on radiological data and the d radiologist's judgment. We defined abnormal cytological and biochemical indexes as below the lower limit and above the upper limit of reference range, as appropriate; e.g. hypoalbuminemia was defined as the serum albumin level lower than 35 g/L, and hypohemoglobinemia was defined as the hemoglobin level lower than the lower limit of the normal reference range (≥ 120 g/L in males and ≥ 110 g/L in females), similar as for others.

### Statistical analysis

Shapiro–Wilk test was used to evaluate the normal distribution status of the continuous variables, and their differences (age, BMI, time from injury to DUS scan, hospital stay) between patients with and without DVT were evaluated by Student *t* test or Whiteny U-test, as appropriate. Categorical variables (sex, prevalence of any comorbidity, smoking, drinking, ASA and the category for laboratory markers) were tested by the *Chi-square* test or Fisher’s exact test, depending on the proportion of the theoretical value in boxes less than 5.

Variables tested with statistical level at *P* < 0.1 in the univariate analyses were further adjusted in the multivariate logistic regression model to investigate their association with DVT, using the stepwise backward elimination method. In the final model, variables that satisfied the significant level *P* < 0.10 were retained, and the correlation magnitude was denoted using odds ratio (OR) and corresponding 95% confidential interval (95%CI). The statistical significance level for all analyses was set as *P* < 0.05. Hosmer–Lemeshow test was performed to evaluate the goodness-of-fit of the final model, with *P* > 0.05 suggesting the acceptable result. Further, the degree of the goodness-of-fit was evaluated by Nagelkerke *R*^2^ value, with larger value indicating the superior result. Given the differences in characteristics of demographics and injury between patients with femoral neck fractures and those with intertrochanteric fractures, we conducted the subgroup analysis stratified by fracture location (femoral neck or intertrochanteric fracture) by repeating the logistics regression analysis with covariates above tested significant in univariate analyses for adjustment. SPSS24.0 (IBM, New York, USA) was used to perform all analyses.

## Results

### General information of patients

This study included 857 eligible patients, including 590 (68.9%) males and 267 (31.1%) females, and their average age was 45.2 ± 10.9 years. 56.0% (480/857) of the fractures were caused by the high or medium-impact mechanism. According to fracture location, 599 (69.9%) were classified as femoral neck fracture and 258 (30.1%) as intertrochanteric fracture. The average time from injury to DUS examination was 4.6 ± 3.6 days, and to the definite operative procedure was 5.2 ± 3.9 days.

**Fig. 1:**
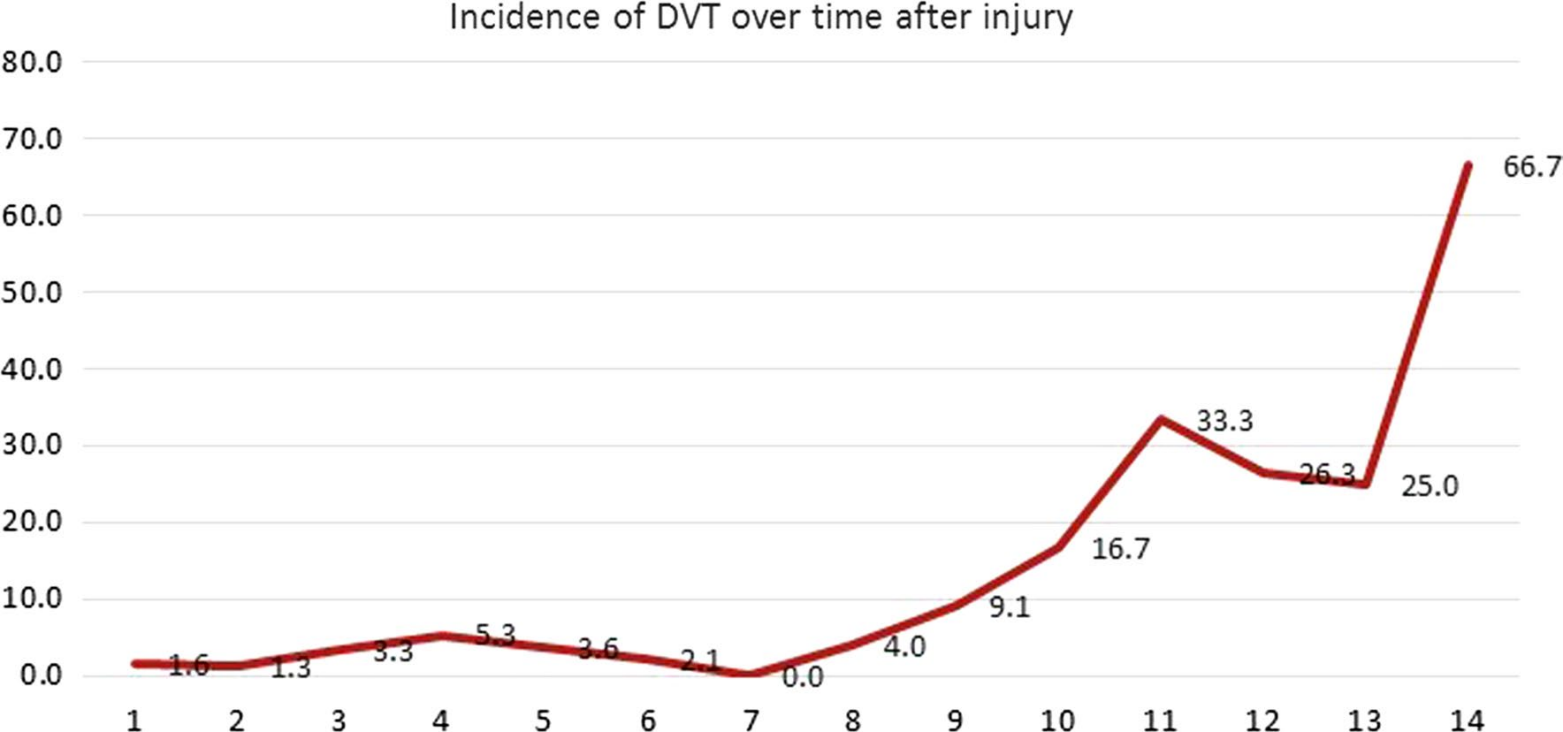
Flowchart of the selection of young and middle-aged hip fractures

Fifty-one patients were diagnosed with preoperative DVTs, suggesting an overall incidence rate of 6.0% (95% CI, 4.4–7.5%). There were 21 patients in the proximal DVT group and 30 in the distal DVT group, with respective incidence rate of 2.5% (95% CI, 1.4–3.5%) and 3.50% (95% CI, 2.3–4.7%). Among DVT patients, the time from injury to DUS scan was 8.6 ± 4.7 days, with 39.2% (20/51) DVTs occurring at day 2 to 4, and 45.1% (23/51) at day 11 to 14 after injury. There was a significant correlation between incidence of DVT and the time since injury (Pearson’s correlation coefficient: *r* = 0.791; *P* = 0.001). The detailed information on DVT occurrence over time was illustrated in Fig. 1. Patients with DVT had a significantly longer hospital stay.

### Univariate analyses

Totally, 97 thrombi were found, predominantly located in distal veins (64.9%, 63/97) and about one third (35.1%, 34/97) in proximal veins. A majority DVTs occurred in the injured side (76.5%, 39/51), 17.6% (9/51) in non-injured side and 5.9% (3/51) in bilateral side. None of DVTs was clinically symptomatic.

Statistical significance was observed for variable regarding age, time from injury to DUS examination, total protein level, albumin level, LDL level, sodium concentration, prevalence of abnormal RBC count, hemoglobin level, hematocrit, and platelet count (all *P* < 0.05, Table 1).

**Table 1:** Univariate analyses of risk factors associated with preoperative DVT following hip fracture

Various	Number (%) of patients with DVT (*n* = 51)	Number (%) of patients without DVT (*n* = 806)	*P* value
Gender			0.066
Male	41 (80.4)	549 (68.1)	
Female	10 (19.6)	257 (31.9)	
Age (year)	48.7 ± 9.4	45.0 ± 10.9	0.018
BMI (kg/m^2^)			0.769
≤ 23.9	31 (60.8)	503 (62.4)	
24–27.9	17 (33.3)	240 (29.8)	
≥ 28	3 (5.9)	63 (7.8)	
Fracture type			0.076
Femoral neck fracture	30 (58.8)	569 (70.6)	
Intertrochanteric fracture	21 (41.2)	237 (29.4)	
Injury mechanism			0.028
High or medium-impact	21 (41.2)	459 (56.9)	
Low-impact	30 (58.8)	347 (43.1)	
Time from injury to DUS (day)	8.6 ± 5.8	3.3 ± 3.1	0.001
Total hospital stays (day)	21.1 ± 9.3	13.4 ± 8.5	0.001
Hypertension	12 (23.5)	170 (21.1)	0.680
Diabetes mellitus	8 (15.7)	80 (9.9)	0.189
Cerebrovascular disease	5 (9.8)	60 (7.4)	0.537
Heart disease	3 (5.9)	45 (5.6)	0.928
Lung disease	3 (5.9)	40 (5.0)	0.770
History of liver disease	3 (5.9)	21 (2.6)	0.169
History of surgery	9 (17.6)	174 (21.6)	0.505
Place of residence			0.124
Rural	39 (76.5)	532 (66.0)	
Urban	12 (23.5)	274 (34.0)	
Cigarette smoking	5 (9.8)	122 (15.1)	0.299
Alcohol consumption	18 (35.3)	202 (25.1)	0.105
ASA scoring			0.749
I–II	44 (86.3)	682 (84.6)	
III–IV	7 (13.7)	124 (15.4)	
Total protein (< 60 g/L)	21 (41.2)	197 (24.4)	0.008
Albumin (< 35 g/L)	28 (54.9)	206 (25.6)	0.001
ALT (> upper limit)	12 (23.5)	125 (15.5)	0.130
AST (> upper limit)	9 (17.6)	113 (14.0)	0.472
HCRP (> 8 mg/L)	44 (86.3)	622 (77.2)	0.130
LDH (> 250 U/L)	26 (51.0)	244 (30.3)	0.002
Sodium (< 137 mmol/L)	31 (60.8)	241 (29.9)	0.001
FBG (**> **6.1 mmol/L)	22 (43.1)	289 (35.9)	0.294
UA (> upper limit)	1 (2.0)	61 (7.6)	0.134
WBC (> 10 * 10^9^/L)	13 (25.5)	252 (31.3)	0.387
Neutrophils count (> 6.3 * 10^9^/L)	25 (49.0)	420 (52.1)	0.668
Lymphocyte count (< 1.8 * 10^9^/L)	20 (39.2)	309 (38.3)	0.900
RBC (< lower limit)	35 (68.6)	298 (37.0)	0.001
HGB (< lower limit)	26 (51.0)	283 (35.1)	0.022
HCT (> upper limit)	44 (86.3)	446 (55.3)	0.001
Platelet (> upper limit)	19 (37.3)	91 (11.3)	0.001
RDW (> upper limit)	3 (5.9)	36 (4.5)	0.677
PDW (< lower limit)	8 (15.7)	79 (9.8)	0.402
D-dimer (> 0.5 mg/L)	39 (76.5)	529 (65.6)	0.112

*DVT* deep venous thrombosis, *BMI* body mass index, *ASA* American Society of Anesthesiologists, *DUS* Duplex ultrasonography, *LDH* lactic dehydrogenase, *WBC* white blood cell, *RBC* red blood cell, *HCT* hematocrit, *ALT* alanine transaminase, *AST* aspartate aminotransferase, *UA* uric acid, *RDW* red cell distribution width, *PDW* platelet distribution width, *FBG* fasting blood glucose

### Multivariate analyses for the DVT

In the multivariate model, age (OR, 1.04; 95% CI, 1.01–1.07), time to DUS (OR, 1.26; 95% CI, 1.18–1.34), elevated LDH level (OR, 1.45; 95% CI, 1.05–2.02), lower sodium concentration (OR, 2.56; 95% CI, 1.29–5.05) and higher HCT (OR, 4.11; 95% CI, 1.60–10.55) (Table 2). The goodness-of-fit of the final model was acceptable (*X*^2^ = 3.801, *P* = 0.875 for Hosmer–Lemeshow test, and Nagelkerke *R*^2^ = 0.343).

**Table 2:** Factors independently associated with the preoperative DVT after young and mid-aged hip fracture patients

Variable	OR and 95% CI	*P* value
Age (increment of each year)	1.04 (1.01–1.07)	0.020
Gender	1.84 (0.96–3.50)	0.065
Time from injury to DUS (increment of each day)	1.26 (1.18–1.34)	0.001
LDH (> 250 U/L)	1.45 (1.05–2.02)	0.026
Sodium (< 137 mmol/L)	2.56 (1.29–5.05)	0.007
WBC (> 10 * 10^9^/L)	0.70 (0.48–1.01)	0.056
HCT (> upper limit)	4.11 (1.60–10.55)	0.003

*DUS* duplex ultrasonography, *LDH* lactic dehydrogenase, *WBC* white blood cell, *HCT* hematocrit

### Subgroup analysis

The subgroup analyses stratified by fracture locations showed that time to DUS was significantly associated with preoperative DVT both for femoral neck fracture (OR, 1.20; 95% CI, 1.10–1.30) and intertrochanteric fracture (OR, 1.42; 95% CI, 1.25–1.62). Other significant variables were age (OR, 1.09; 95% CI, 1.02–1.15), elevated LDH level (OR, 1.89; 95% CI, 1.23–2.91) and lower sodium concentration (OR, 2.35; 95% CI, 1.27–5.67) for femoral neck fracture, and male (OR, 2.36; 95% CI, 1.27–6.02) and higher HCT (OR, 4.27; 95% CI, 1.31–13.14) for intertrochanteric fracture. WBC was not a significant factor for preoperative DVT, for either fracture type (Table 3).

**Table 3:** Subgroup analyses stratified by fracture location for factors independently associated with the preoperative DVT

Variable	OR and 95% CI	*P* value
Femoral neck fracture		
Time from injury to DUS (increment of each day)	1.20 (1.10–1.30)	< 0.001
Age (increment of each year)	1.09 (1.02–1.15)	0.006
LDH (> 250 U/L)	1.89 (1.23–2.91)	0.004
Sodium (< 137 mmol/L)	2.35 (1.27–5.67)	0.008
Intertrochanteric fracture (versus femoral neck fracture)		
Time from injury to DUS (increment of each day)	1.42 (1.25–1.62)	< 0.001
Gender (male versus female)	2.36 (1.27–6.02)	0.013
WBC (> 10 * 10^9^/L)	0.55 (0.29–1.07)	0.076
HCT (> upper limit)	4.27 (1.31–13.14)	0.002

*DVT* deep venous thrombosis, *DUS* duplex ultrasonography, *LDH* lactic dehydrogenase, *WBC* white blood cell, *HCT* hematocrit, *OR* odd ratio, *CI* confidential interval

## Discussion

Extensive knowledge about the epidemiologic characteristics of DVT after hip fracture in the young and mid-aged patients is of crucial importance in prevention and management this complication. However, this remains a hanging issue. In this study, we used prospectively collected data in this specific subgroup to conduct a retrospective secondary analysis, regarding the over incidence rate of DVT and that with time after injury, the detailed DVT location, and the factors that could predict the DVT occurrence. By far as we know this is the first study focused on the subgroup of young and mid-aged patients to address the DVTs, and these findings could be beneficial in informed preventive of DVT and optimized management of hip fracture.

Compared to elderly patients, incidence of preoperative DVT after hip fracture was significantly lower in the young and mid-aged patients, about 1/7 to 1/4 of that of the former group [9–11]. This difference may be related to the unfavorable vascular and blood flow conditions, highly prevalent comorbidities, and the undesirable systemic stress/inflammatory response to trauma in the elderly patients [9, 11]. In this study, we found the relatively high incidence rate of proximal DVT, which, partly, can be explained by the theory related to fracture location, that is the more proximal the fracture site, the higher the incidence of proximal DVT [12]. The mechanism of higher-energy impact in the younger patients was also an important contributing factor for this finding.

Consistent with most but not all the previous findings, DVTs were predominantly located in the veins of injured extremity, and the proportion is about 76.5%, in range of the previously reported figures [10–12]. Sevitt et al. [15] reported an extreme case in their study focusing on injured and burned patients, that the DVTs were diagnosed solely in the injured extremity. We thought this result might be related to the nature and severity of original injury, and the burned and injured conditions made hemodynamic disorders more severe than hip fracture in this study. Despite the huge difference in DVT distribution, examination the uninjured extremity for potential DVTs remains not negligible, and in practice this exactly may be an important cause of a missed diagnosis.

In theory, the blood coagulation status is dynamically changing over time after a hip fracture, which, together with changing immune/inflammatory response to trauma, contributes to the observed highly variable incidence rates of DVT. In this study, we summarized respective incidence rate of DVT over time and found the significant positive relationship between them, and the multivariate analysis showed this relationship was independent of demographics and several biochemical markers, and significant for both femoral neck fracture and intertrochanteric fracture. We also observed that from 10th day after fracture, incident DVTs were markedly increased, and the corresponding rate was 30.6% in average, even up to 66.7% at the 14th day. Therefore, despite the relatively low DVT incidence (6.0%), patients who are transferred at a significantly delayed time post-injury (particularly, above 10 days) should be classified as the key high-risk group. To the best of our knowledge, this finding was the firstly reported. Hence, considering the tertiary referral setting of our institution, it is necessary to know about fracture occurrence time and thromboembolic agents use at the treating hospitals before they are transferred.

Besides the delay to DUS examination, we have identified age, abnormal LDH, sodium and HCT level as factors predicting DVT. Despite a setting of young and mid-aged patients, age remains to be an independent factor for DVT, and the effect size in this study (OR, 1.04) was comparable to that (range, 1.03–1.07) in the elderly hip fracture patients [9, 11]. Interestingly, the age seems non-significant when subgroup analysis was restricted to intertrochanteric fracture. These findings suggest that the influential effect of age on blood vessel endothelium and hemodynamics may be persistent, but was more likely to affect DVT following femoral neck fracture. The lower sodium concentration means the remediation of the systemic balance of fluid and serum sodium after the blood loss (including overt and occult) in hip trauma, but also reflects the initial trauma-fracture severity and the secondary blood hypercoagulability to a large extent. Its relationship with DVT has been identified in a cohort study using the large administrative database, that hyponatremia was associated with 1.39-fold increased risk of postoperative DVT for any surgical specialties, independent of confounders [16]. The elevated HCT level and hence the hyperviscosity was associated with vascular disturbance [17, 18], potentially contributing the risk of thrombosis; and indeed, lowering the hematocrit by phlebotomy had been demonstrated effective in reducing DVT risk [19].

It is a surprising finding that plasma D-dimer level was not identified to be associated with DVT, neither in univariate nor multivariate analysis, contradictory to most but not all previous studies. We thought this can be explained by the several facts. First, we used a conventional cut-off value (0.5 mg/L) for dichotomizing D-dimer level, which may be not adequately predisposed to predicting preoperative DVT; in contrast, cut-off values of D-dimer level of 1.1–1.8 mg/L were identified to have adequate power to discriminate the DVT events [20–22]. Second, the included patients are young and mid-aged, in whom the D-dimer level was lower than in elderly patient, and the sensitivity to predicting DVT is reasonably low. Third, despite a non-significant result in the univariate, we observed the trend to approaching to significance and we thought the inadequate sample especially the limited DVTs contributed a large part. The future study should still focus on the age-adjusted D-dimer level for its significance in predicting or diagnosing the thromboembolism events.

The methodological strengths of this study included the use of prospectively collected data, the first report of the incidence rate of DVT on a daily basis after hip fracture in a setting of underappreciated group, and multiple potential variables for adjustment. Several limitations should be addressed. First the setting of a tertiary referral of this hospital may a major source of selection bias, because patients surgically treated can have more complex medical conditions or severer injury. Hence, the generalizability of these findings is discounted. Second, we are unable to know about the detailed use of thromboembolic agents at the initial hospitals, which can significantly affect the incident DVTs. Third, as with every multivariate analysis, there remain the residual confounding effects due to the unconsidered, unmeasured or unavailable variables, such as use of glucocorticoids or the number of cigarettes per day for smokers. Fourth, this is a cross-sectional study rather a cohort study, thus not allowing obtaining causal relationship between factors and incident DVT.

In summary, we found the relatively low incidence rate of preoperative DVT after hip fracture in a specific group of young and mid-aged patients. Despite this, patients with delayed transfer or admission, especially above 10 days after fracture, should be considered as particularly high-risk group and be given targeted management. Age, time to DUS, elevated LDH level, lower sodium concentration and higher HCT level were identified to be independently associated with DVT. Although most not modifiable, they could be beneficial in informed preventive of DVT and optimized management of hip fracture.

## Data Availability

All the data will be available upon motivated request to the corresponding author of the present paper.

## Abbreviations

DVT: Deep venous thrombosis
DUS: Duplex ultrasonography
STROCSS: Strengthening the Reporting of Cohort Studies in Surgery
BMI: Body mass index
RBC: Red blood cell
WBC: White blood cell
ASA: American Society of Anesthesiologists
LDH: Lactic dehydrogenase
HCT: Hematocrit
ALT: Alanine transaminase
AST: Aspartate aminotransferase
UA: Uric acid
RDW: Red cell distribution width
PDW: Platelet distribution width
FBG: Fasting blood glucose
OR: Odd ratio
CI: Confidential interval
SD: Standard deviation
